# Patients’ attitude towards a sham-controlled trial on pulmonary vein isolation in atrial fibrillation

**DOI:** 10.1007/s00392-021-01959-z

**Published:** 2021-10-28

**Authors:** Tobias Uhe, Samira Beimel, Romy Langhammer, Tina Stegmann, Gerhard Hindricks, Ulrich Laufs, Nikolaos Dagres, Rolf Wachter

**Affiliations:** 1 Klinik und Poliklinik für Kardiologie, Universitätsklinikum Leipzig, Liebigstraße 20, 04317 Leipzig, Germany; 2 Abteilung für Rhythmologie, Herzzentrum Leipzig, Leipzig, Germany

**Keywords:** Atrial fibrillation, Catheter ablation, Pulmonary vein isolation, Sham-controlled trial

## Abstract

**Background:**

The interpretation of recent trials on pulmonary vein ablation (PVI) for the treatment of atrial fibrillation (AF) is hampered by the lack of blinding and sham controls. The feasibility of a sham-controlled trial has been questioned. We aimed to assess the attitude of potential participants regarding a sham-controlled trial in a common AF-patient population planned for PVI.

**Methods:**

Patients in two tertiary care centres planned for PVI were asked for their current AF symptoms using the Atrial Fibrillation Effect on QualiTy of Life (AFEQT) questionnaire 1 day before catheter ablation. Subsequently, the study design of a hypothetical sham-controlled PVI-study was introduced, and patients were asked for their agreement in participation. Telephone follow-up of the AFEQT questionnaire was conducted 3 months after PVI.

**Results:**

One hundred and ninety-six patients (mean age 64 ± 11 years, 63% male) were included. Seventy-nine (40%) patients expressed their agreement to participate in the hypothetical sham-controlled trial. An additional 7% agreed to participate if a cross-over option after three months was offered. Agreement rate was similar in patients with first and Redo-PVI and minimal, moderate or severe symptoms. Mean overall AFEQT at baseline was 55 ± 19 and improved by 25 ± 20 points after 3 months (*p* < 0.001 versus baseline).

**Conclusion:**

With a participation rate of 40% in potential study participants, a sham-controlled trial for pulmonary vein isolation seems feasible. Patient-reported symptom relief after pulmonary vein isolation is in accordance with previous randomized open studies. The benefit of PVI should be rigorously evaluated in a sham-controlled trial.

**Graphic abstract:**

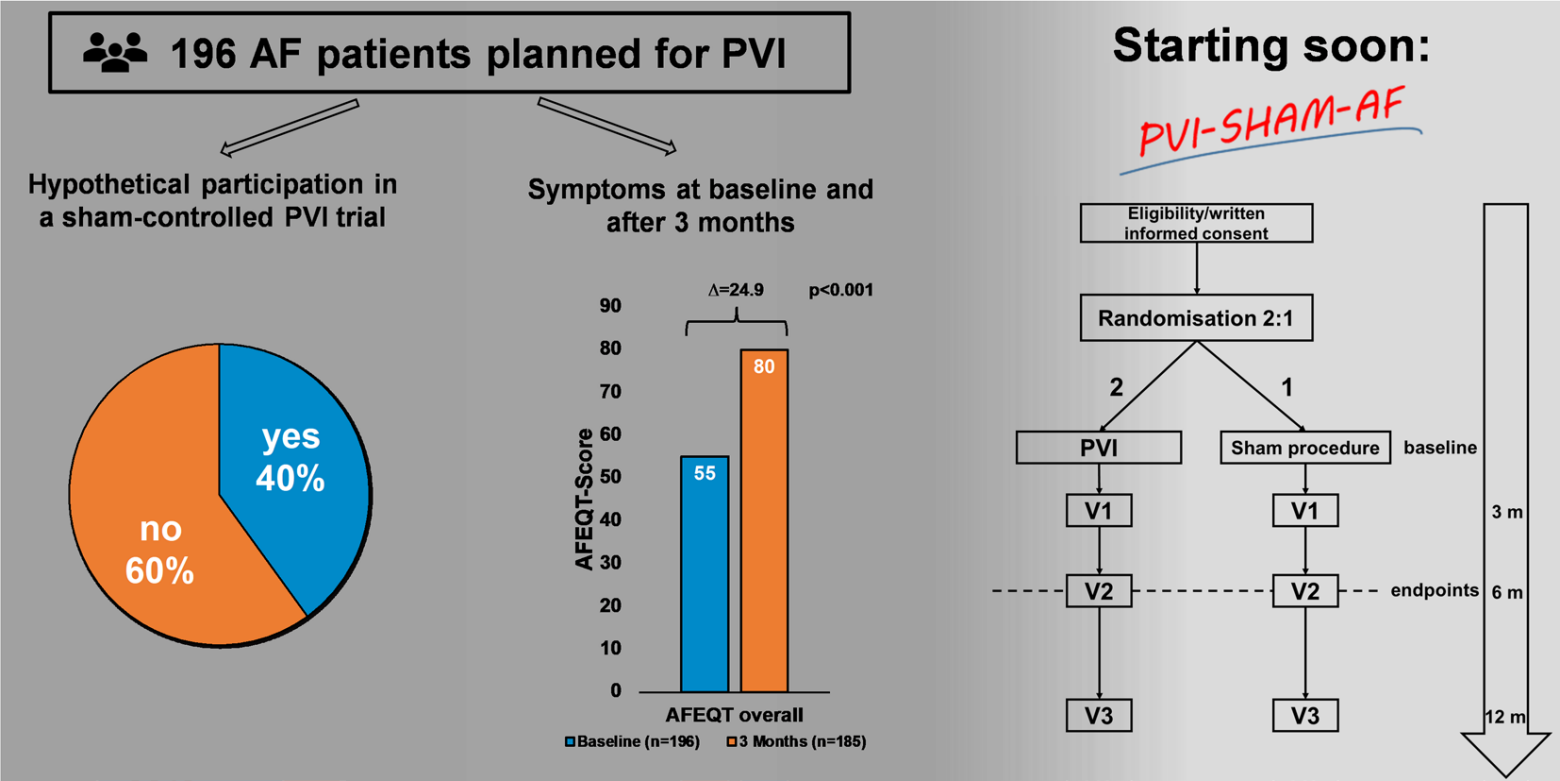

**Supplementary Information:**

The online version contains supplementary material available at 10.1007/s00392-021-01959-z.

## Introduction

Atrial fibrillation (AF) is the most common arrhythmia and impairs public health. Prevalence and incidence are expected to increase significantly within the next years, especially in the elderly population [1, 2].

The guidelines for the diagnosis and management of atrial fibrillation published in 2020 by the European Society of Cardiology (ESC) introduced the new treatment pathway “ABC” (‘A’ Anticoagulation/Avoid stroke, ‘B’ Better symptom management, ‘C’ Cardiovascular and Comorbidity optimization) [3]. In this therapy concept, pulmonary vein isolation (PVI) is an important component of better symptom control. Symptom control by any medical intervention is confounded by a relevant placebo effect [4]. For example, this was recently shown for percutaneous coronary intervention (PCI) in the ORBITA trial or renal denervation in the SYMPLICITY HTN-3 trial [5, 6].

Approximately 24,000 PVI procedures are performed in Germany every year and the success rate of PVI regarding maintenance of sinus rhythm is reported to be 60–70% [7–10]. Complications occur in approx. 4–14% of the patients [3, 8, 11, 12]. PVI improves the prognosis in patients with heart failure and reduced ejection fraction [13]. In all other patients, neither stroke, death nor cardiac hospitalization are prevented by PVI [14]. Quality of life (QoL), as measured by the Atrial Fibrillation Effect on QualiTy of Life (AFEQT) questionnaire, was significantly improved by PVI in the recently published CABANA trial, but the true ‘placebo-controlled’ effect of PVI on QoL is unknown [15]. We therefore aim to conduct a double-blinded randomized and sham-controlled trial on symptomatic AF patients.

The *Achilles’ heel* in the conduction of sham-controlled randomized controlled trials (RCT) of invasive procedures is the recruitment process and patients’ participation rate [16]. The feasibility of a sham-controlled trial for symptomatic benefit of PVI in AF patients has been questioned as the number of patients accepting randomization to ‘true-PVI’ versus ‘sham-PVI’ may be very low. We therefore assessed patients’ attitude towards a randomized trial as the main hurdle in starting such a trial. We therefore conducted an explorative survey in symptomatic AF patients planned for PVI on their willingness to participate in a randomized, sham-controlled PVI trial. Furthermore, we aimed to ascertain the characteristics of this patient collective and their symptomatic benefit from the PVI after 3 months to define a primary symptomatic endpoint for a sham-controlled PVI-trial.

## Methods

From January to August 2020, we enrolled patients with paroxysmal or persistent AF planned for PVI at the Clinic and Policlinic for Cardiology, University of Leipzig and at the Department of Electrophysiology at the Heart Centre Leipzig. The Ethics committee of the University of Leipzig approved the study and all study participants confirmed written consent.

At enrolment, patients underwent medical history, physical examination, 12-lead-electrocardiogram (ECG) and routine laboratory assessment according to local standards. Cardiac imaging was performed either using echocardiography or cardiac magnetic resonance imaging (MRI) according to local standards and current guidelines [17, 18].

### Assessment of patients’ willingness to participate and quality of life

One day before catheter ablation, we asked patients to give informed consent for a survey on participation in a sham-controlled trial. The patient information is available with this manuscript (Supplemental material S2). All patients were interviewed by the same person. At any time, patients were aware to receive the PVI they were planned for at the next day. Patients who refused participation were asked for their rationale in a standardized questionnaire. Additionally, these patients were questioned if they would agree to participate if a cross-over option to a guaranteed PVI was offered after three months.

Subsequently, QoL was assessed using the AFEQT questionnaire. This AF-specific score consists of 21 items which can be responded to on a Likert Scale from 1 (“Not at all”) to 7 (“Extremely”). The questionnaire consists of the following subscales: Symptoms, Daily Activities (DA), Treatment Concern and Treatment Satisfaction. The overall score and the respective subscale scores range from 0 to 100 where 0 represents the greatest impairment of QoL due to AF and 100 indicates no disability or limitation [19].

Patients were divided into the following groups according to their symptom severity: severely symptomatic (AFEQT < 70), moderately to mildly symptomatic (AFEQT 70–89) and minimally symptomatic to asymptomatic (AFEQT ≥ 90) [15].

Procedural characteristics of the PVI such as ablation duration, ablation technique, additional ablations and adverse events were obtained along with medication at discharge.

### Follow-up

Telephone follow-up was conducted 3 months after PVI and structured as follows: first, patients were asked again whether they could imagine participating in a sham-controlled study. Second, current medical status was obtained including AF or stroke hospitalization, changes in medication and subjective AF recurrence. Third, AF-specific symptoms and QoL were re-assessed using AFEQT-score.

### Statistical analysis

Continuous variables are given as mean ± standard deviation if normally distributed or as median and interquartile range of the 25th and 75th centile if not normally distributed. Categorical variables are shown as absolute numbers (%). Normally distributed data were compared by student’s *t* test, not normally distributed data by Mann–Whitney *U* test and categorical variables by Chi-square test. AFEQT overall and subscale differences were calculated using ANOVA. Univariate correlation of categorical baseline characteristics with willingness to participate was calculated using Chi-square and Eta statistics. Multivariable correlation was calculated with binary logistic regression. All tests were performed with SPSS Statistics 25.0 (IBM, Chicago, Illinois, USA). *p* values < 0.05 were considered to be significant.

## Results

### Study population

**Fig. 1:**
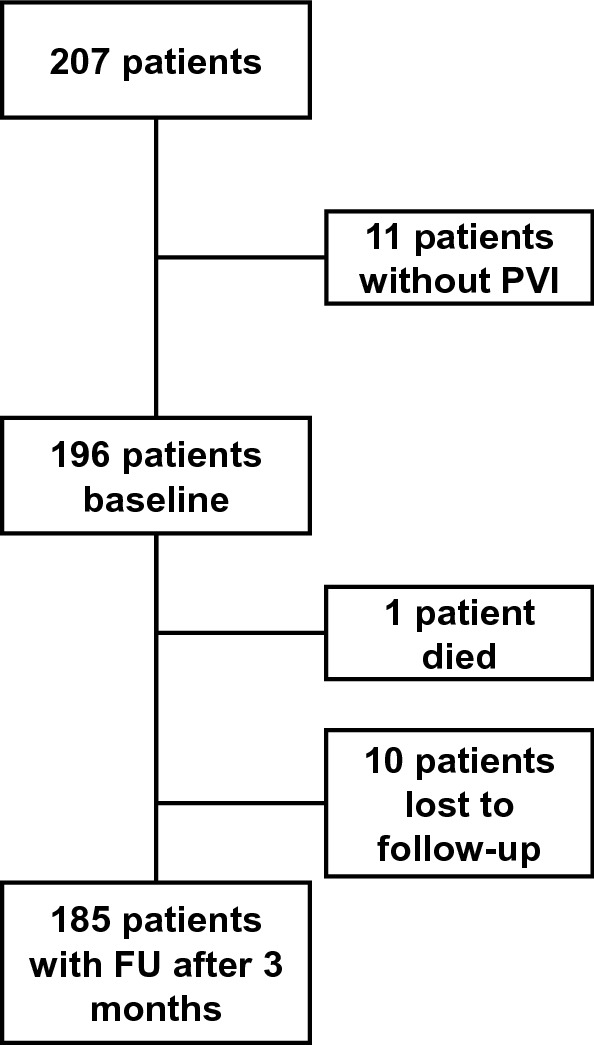
Study flowchart. *PVI* pulmonary vein isolation; *FU* follow-up. All participants lost to follow-up declined to be interviewed again by phone after 3 months

Between January and August 2020, we enrolled two-hundred-seven patients planned for catheter ablation of AF. Two patients had atrial flutter and underwent ablation of the cavotricuspid isthmus instead of PVI. Nine patients did not receive PVI due to other medical reasons (e.g., acute infections). Baseline information was obtained for 196 patients. Telephonic follow-up was conducted in 185 patients after three months. One patient died while ten patients were lost to follow-up. The study flowchart is shown in Fig. 1.

### Baseline characteristics

Baseline characteristics are shown in Table 1 and compared for patients undergoing first PVI versus patients undergoing a Redo-PVI. Patients with previous PVI less often had diabetes, a longer history of AF, more often AF in admission ECG and larger left atria.

**Table 1:** Baseline characteristics

Baseline characteristics	First PVI (*n* = 144)	Redo-PVI (*n* = 52)	*p*
Age (y)	65 [65; 72]	69 [61; 74]	0.10
Male gender	91 (63)	33 (64)	0.97
BMI (kg/m^2^)	29.1 ± 4.7	29.5 ± 4.6	0.66
Type of AF			
Paroxysmal	67 (47)	19 (37)	0.21
Persistent	77 (53)	33 (63)	0.18
Time since first diagnosis (months)	47.9 ± 61.1	96.8 ± 85.0	< 0.001
EHRA-Score	2 [2; 3]	2 [2; 3]	0.96
EHRA I	5 (4)	1 (2)	1.00
EHRA II	87 (60)	33 (63)	0.70
EHRA III	52 (36)	18 (35)	0.85
CHA_2_DS_2_-VASc-Score	3 [1; 4]	3 [2; 4]	0.859
Comorbidities			
Hypertension	116 (81)	43 (83)	0.74
Heart failure	25 (17)	9 (17)	0.99
Diabetes mellitus	37 (26)	4 (8)	0.01
Coronary heart disease	41 (29)	10 (19)	0.19
Prior myocardial infarction	11 (8)	2 (4)	0.52
Prior Stroke or TIA	13 (9)	9 (17)	0.11
Peripheral artery disease	5 (4)	1 (2)	1.00
Hyperlipoproteinemia	83 (58)	33 (64)	0.46
12-Lead ECG at admission			
*Rhythm*			0.03
Sinus rhythm	76 (53)	16 (31)	
Atrial fibrillation/flutter	65 (45)	32 (61)	
Other	3 (2)	4 (8)	
Heart rate	75.6 ± 21.2	93.4 ± 26.2	< 0.001
Laboratory values			
Haemoglobin (mmol/l)	8.8 ± 1.1	9.0 ± 0.9	0.37
Creatinine (µmol/l)	90.1 ± 27.7	87.2 ± 18.5	0.48
Cardiac imaging			
Left ventricular ejection fraction (%)	56 ± 10	55 ± 8	0.56
Left atrium area (cm^2^)	27.3 ± 7.2	30.4 ± 6.3	0.02
Medication			
Beta blockers	126 (88)	49 (94)	0.18
ACEI/ARB/ARNI	97 (67)	35 (67)	0.72
Diuretics	52 (36)	23 (44)	0.32
Mineralocorticoid receptor antagonists	27 (19)	4 (8)	0.06
Calcium channel blockers	38 (26)	17 (33)	0.39
Other antihypertensive agents	15 (10)	3 (6)	0.41
Flecainide	20 (14)	7 (14)	0.94
Amiodarone	16 (11)	4 (8)	0.60
Dronedarone	3 (2)	0 (0)	0.58
Propafenone	1 (1)	0 (0)	1.00
Digitalis	8 (6)	4 (8)	0.74
Ivabradine	2 (1)	0 (0)	1.00
Oral anticoagulation	132 (92)	49 (94)	0.76
Antiplatelets	7 (5)	0 (0)	0.19
Statins	55 (38)	25 (48)	0.21

Values are given as mean ± standard deviation, median and interquartile range or *n* (%)

*PVI* pulmonary vein isolation; *BMI* body mass index; *AF* atrial fibrillation; *EHRA* European Heart Rhythm Association; *TIA* transient ischemic attack; *ECG* electrocardiogram; *ACEI* angiotensin converting enzyme inhibitor; *ARB* angiotensin receptor blocker; *ARNI* angiotensin receptor neprilysin inhibitor

### Willingness to participate in sham vs. pulmonary vein isolation (PVI) study and reasons to refuse

**Fig. 2:**
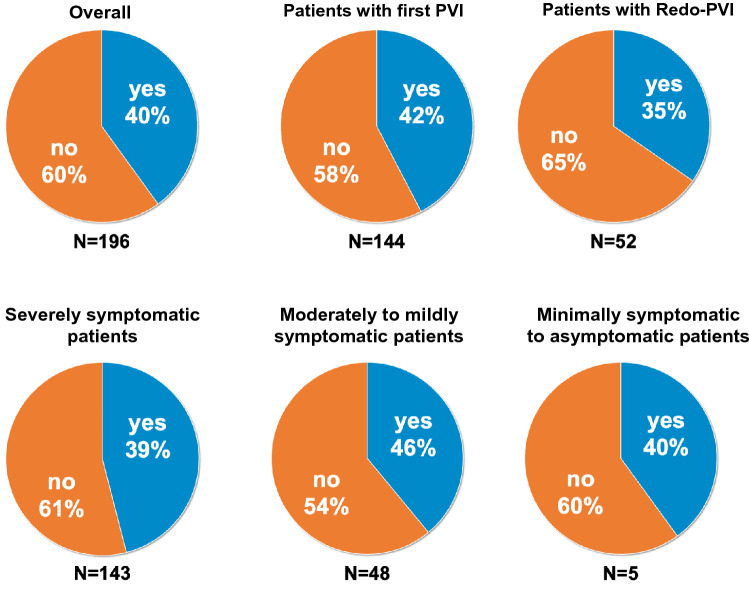
Rate of hypothetical participation in a sham-controlled pulmonary vein isolation (PVI) trial overall, in patients undergoing their first PVI, patients undergoing a Redo-PVI and in different symptom severity groups assessed by AFEQT score. *AFEQT* Atrial Fibrillation Effect on QualiTy of Life questionnaire

At baseline 79 (40%) out of 196 patients reported their willingness to participate in a sham-controlled PVI-trial. An additional 7% (*n* = 14) agreed to participate in case of a cross-over option to a guaranteed PVI after three months. The participation rate depending on symptom severity was 39% in severely symptomatic patients (55/143), 46% in moderately to mildly symptomatic patients (22/48) and 40% in minimally symptomatic to asymptomatic patients (2/5) and did not differ significantly in between the groups (*p* = 0.67, Fig. 2). The main reason to decline participation was the fact that the patients were admitted by their treating physician to receive a PVI and did not want this decision to be changed (Supplement Material S1).

### Predictors for higher participation rate

We found no significant differences in willingness to participate between patients undergoing their first PVI and those undergoing Redo-PVI (42% vs. 35%; *p* = 0.33). Of all baseline characteristics, only persistent AF (OR 2.0, 95% CI [1.1; 3.5]), male gender (OR 2.6, 95% CI [1.4; 4.9]) and previous stroke or transient ischemic attack (TIA) (OR 4.7, 95% CI [1.8; 12.6]) were associated with a higher participation rate in univariate analysis.

In multivariable analysis including variables significantly associated with a higher participation rate in univariate analysis, higher participation rate was associated with male gender and previous stroke/TIA but not with persistent AF (Table 2).

**Table 2:** Multivariable analysis of predictors of participation in a sham-controlled pulmonary vein isolation (PVI) trial

Parameter	Odds ratio [95% CI]	*p v*alue
Male gender	2.57 [1.3; 5.22]	0.006
Previous stroke/TIA	4.76 [1.71; 13.22]	0.003

*TIA* transient ischemic attack

Variables significantly associated with a higher participation rate in univariate analysis, i.e., male gender, persistent atrial fibrillation and previous stroke/TIA were included in the model

### Quality of life at baseline and after 3 months

**Fig. 3:**
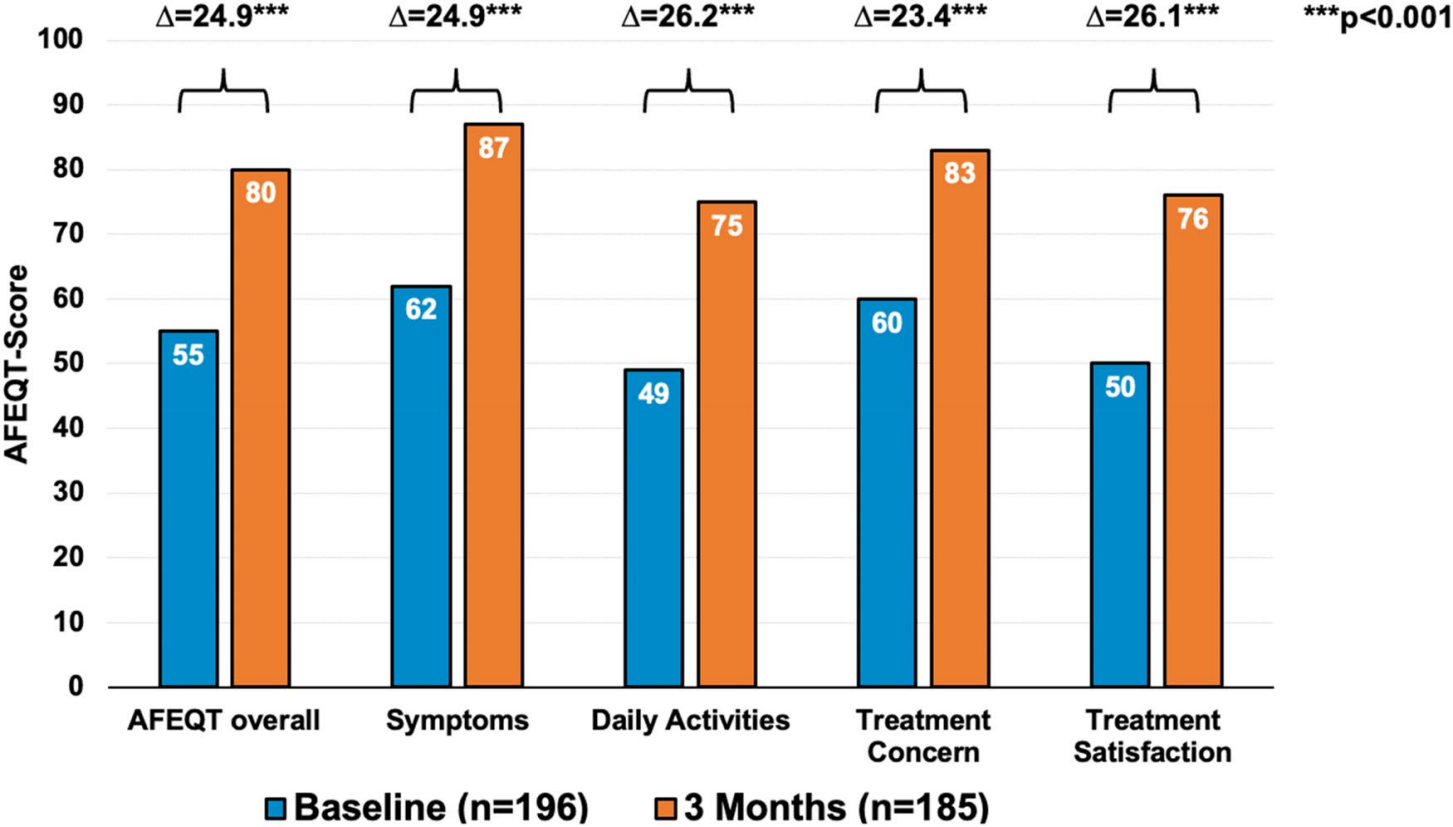
Mean AFEQT score overall and AFEQT score subscales at baseline (blue columns) compared to 3 months post-PVI (orange columns). Differences were calculated using ANOVA. *AFEQT* Atrial Fibrillation Effect on QualiTy of Life questionnaire, *PVI* pulmonary vein isolation

Mean overall AFEQT score at baseline was 55 ± 19 points and improved by 25 ± 20 points (*r* = 0.78, *p* < 0.001). AFEQT component score values improved by 25 ± 27 points (*p* < 0.001) for symptoms, by 26 ± 27 points (*p* < 0.001) for DA and by 23 ± 22 points (*p* < 0.001) for treatment concern. Treatment Satisfaction score improved by 26 ± 30 points (*p* < 0.001) (Fig. 3). Patients undergoing first PVI did neither differ in their baseline overall AFEQT score (56 ± 20 vs. 53 ± 19; *p* = 0.27) nor in their overall AFEQT score improvement (25 ± 19 vs. 26 ± 22; *p* = 0.75) from patients undergoing a Redo-PVI.

**Fig. 4:**
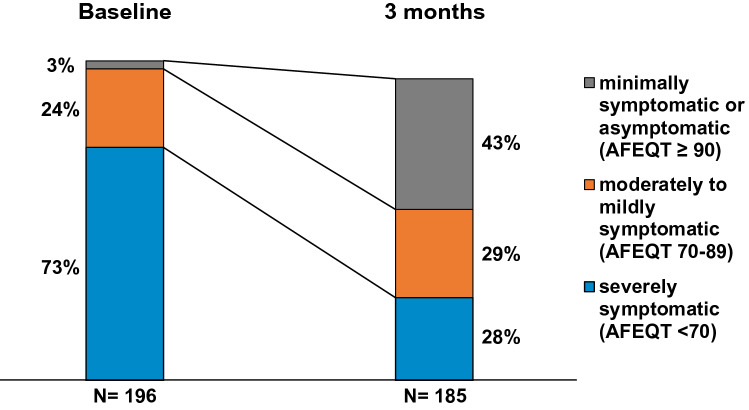
Changes in overall AFEQT symptom severity groups from baseline to 3 months. *AFEQT* Atrial Fibrillation Effect on QualiTy of Life questionnaire

**Fig. 5:**
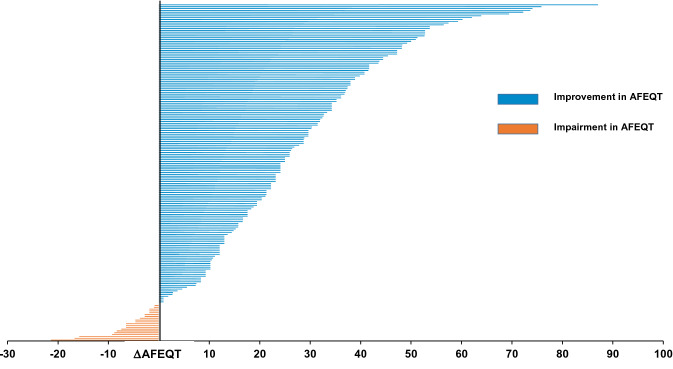
Change in AFEQT overall three months post PVI compared to baseline for individual patients. *AFEQT* Atrial Fibrillation Effect on QualiTy of Life (AFEQT) questionnaire, *PVI* pulmonary vein isolation

Of all 196 patients, 143 patients (73%) were severely symptomatic, 48 patients (24%) were moderately to mildly symptomatic and five patients (3%) were minimally symptomatic or asymptomatic. After 3 months 51 patients (28%) were severely symptomatic, 54 patients (29%) were moderately to mildly symptomatic and 80 patients (43%) were minimally symptomatic or asymptomatic. Figure 4 shows the distribution for different severity subgroups before and three months after PVI. Twenty patients (10%) had a worse AFEQT after three months while 165 (90%) patients improved. Individual AFEQT change is shown in Fig. 5.

## Discussion

### Main findings

We assessed the willingness of AF patients to participate in a sham-controlled PVI-trial and found a participation rate of 40% and up to 47% if a cross-over option to a guaranteed PVI was offered. Most frequently, patients stated to refuse participation because they had been admitted to the hospital especially for PVI and, therefore, claimed the planned procedure to be performed accordingly.

Our study is the first to survey the willingness of AF patients to participate in a sham-controlled PVI trial. The number of patients who declined their participation in randomized sham-controlled trials is usually not listed in detail or difficult to track since several patients may have dropped out for competing reasons, e.g., fulfilling exclusion criteria.

In a multicentre trial assessing the effect of PCI in patients with stable angina pectoris, the rate of patients who declined participation was 23% [6]. Nevertheless, the number of reported eligible patients (*n* = 368) remains questionable low according to the high overall number of PCIs and the recruitment period of more than 3 years in five study sites. In contrast, a detailed study flowchart including a high number of eligible patients has been provided by Desch et al. in a renal sympathetic denervation study in patients with hypertension [20]. The authors reported a participation rate of 52% in patients eligible for randomization, i.e., patients who met all inclusion criteria, which is similar to our results. However, the attitude of patients towards participation in a sham-controlled trial might be higher in diseases with a lower level of suffering, e.g., in hypertension compared to AF and in studies investigating novel treatment methods that are not offered as part of the routine treatment. In this respect, our results provide a conclusive basis for the feasibility of a sham-controlled PVI-trial.

In multivariable analysis, we found only male gender and previous stroke or TIA to be associated with higher participation rates. To date, no other cardiovascular sham-controlled trial has provided information on predictors of participation. Since patient enrolment is a commonly reported difficulty in the conduction of sham-controlled trials, further investigation on the association between patients’ characteristics and a higher participation rate is required to ensure a balanced study collective for such a trial.

### Quality of life after pulmonary vein isolation

We found a significant improvement in QoL in patients with AF three months after PVI. Our results are consistent with other studies that assessed QoL before and after PVI using AFEQT.

Two studies included AF patients with a similar overall AFEQT at baseline and followed them for three months. Both analyses showed an improvement of 23 and 25 points, respectively, in the overall AFEQT score three months after PVI which is in accordance to our results [19, 21].

Several other studies assessed QoL as a primary or secondary endpoint 12 months after catheter ablation: the CAPCOST and EARLY-AF trial observed a mean improvement in overall AFEQT score of 28 and 27 points, respectively, after 1 year [22, 23].

At baseline, patients had a relevantly impaired QoL across all subscales with the greatest impairment in the DA subscale. After three months, all components of the AFEQT score showed similar improvement with the greatest improvement in DA as well. These results are in line with the subanalysis of the catheter ablation group of the CABANA study in this respect [15].

Ninety percent of the patients in our study showed an improved QoL after PVI, which is comparable to the findings of the GOLD AF registry [24].

In the randomized CABANA trial, patients randomised to catheter ablation had a mean improvement in the overall AFEQT score of 17 points after 3 months which is lower than the observed difference in our study [15]. The comparatively smaller increase in QoL after 3 months could be due to a higher AFEQT score at baseline in CABANA, which means that there was less room for improvement in CABANA than in our study (overall AFEQT 63 ± 21 in CABANA compared to 55 ± 19).

We found no difference in QoL improvement after catheter ablation between patients undergoing their first PVI and those undergoing a Redo-PVI which confirms previous reports. Kany et al. reported a similar percentage of patients with symptom improvement or no symptoms, but no detailed assessment of QoL was performed [25]. Similarly, Pezawas et al. did find similar improvements in QoL, measured by the Short-Form Health Survey (SF-12) in patients receiving first or Redo-PVI [26].

### Feasibility of a sham-controlled trial for pulmonary vein isolation

In the last decade, the number of sham-controlled cardiovascular trials has rapidly evolved and the conduction of such trials has become a requirement in the process of certification of novel devices and interventional therapies whenever ethical and feasible [27]. While a trial with sham-controlled design does not correct for all possible causes of bias, such as co-interventions, it is a valid design to assess a possible placebo effect of an intervention [28]. For pulmonary vein isolation, no such trial exists or is currently underway. The most prominent argument by physician is that patients who are highly symptomatic do not consent to randomisation in a sham-controlled trial. Our data clearly show, that 40% of patients scheduled for pulmonary vein ablation would agree to randomisation. Importantly, this number may increase if a timely cross-over possibility after three month is granted. Taken together our data indicate that a sham-controlled trial for PVI should be initiated. It would also allow to estimate which percentage of QoL improvement is attributable to the procedure itself and the placebo effect. Interestingly, patient-reported outcomes such as the AFEQT score are more susceptible to placebo effects than objective outcomes [29].

### Limitations

All patients were aware of the hypothetical character of our pilot study and to receive a PVI as planned during their hospital stay. This limitation is likely to have biased the results regarding patients’ attitude towards participation in a sham-controlled trial and the true participation rate may be lower than 40%. Information on the patients' level of education was not collected, although it is conceivable that a higher level of education was associated with a greater willingness to participate [30].

A crossover possibility after 3 months may be too early and may overestimate the true participation rate. We therefore propose a cross-over possibility after 6 months for the now planned randomized PVI-sham-AF study. This might slightly reduce the number of patients willing to participate in the PVI-sham-AF study.

Moreover, patients were followed up for 3 months only. Consequently, this study only provides information about short-term effects of PVI on QoL. Furthermore, follow-up was conducted by telephone only and no valid data on AF recurrence have been raised. Since the correlation of symptoms with AF recurrence or AF burden is low, these surveys are not essential for the investigation of a symptomatic outcome [31].

Due to the exploratory character of our study and to assess the entire collective of AF patients planned for PVI, we have not predefined any specific inclusion or exclusion criteria. However, the hypothetical participation rate in a sham-controlled trial was similar in severely compared to moderately and minimally symptomatic patients and in patients undergoing their first PVI compared to those undergoing a Redo-PVI and represents therefore a valid measurement for the entire collective.

## Conclusion

The evaluation of the ‘true symptomatic effect’ of pulmonary vein isolation requires the conduction of a sham-controlled randomized trial. With a potential participation rate of 40% among patients with atrial fibrillation planned for catheter ablation, the recruitment for such a trial is feasible. Our findings regarding the quality of life after pulmonary vein isolation are in accordance with previously published studies and provide a solid basis for the calculation of a primary symptomatic endpoint.

## Supplementary Information

Below is the link to the electronic supplementary material.

Supplementary file1 (DOCX 136 KB)
