# Supplementary material for: Patients’ attitude towards a sham-controlled trial on pulmonary vein isolation in atrial fibrillation
Source: Clin Res Cardiol. 2021 Oct 28;111(1):114–23. doi: 10.1007/s00392-021-01959-z (PMC8766391; doi:10.1007/s00392-021-01959-z)
Supplement: Supplementary file 1 — Supplementary file1 (DOCX 136 KB) [file 392_2021_1959_MOESM1_ESM.docx]

# Supplemental material


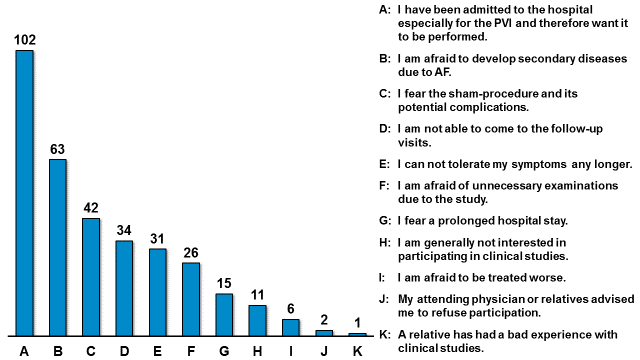


Figure S1: Reasons to refuse participation in a sham-controlled PVI trial. Multiple responses were possible. PVI=pulmonary vein isolation, AF=atrial fibrillation

*
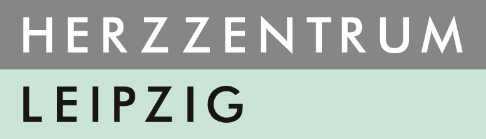

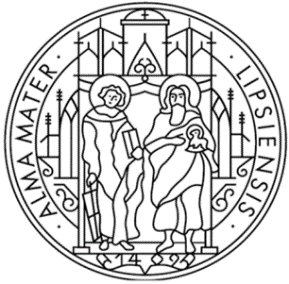
***
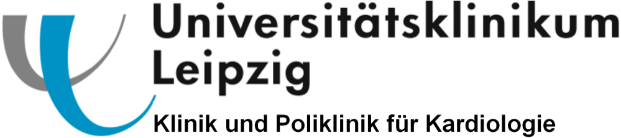
**

**Machbarkeitsanalyse einer sham-kontrollierten Studie zur Pulmonalvenenisolation**

Pilot study to determine feasibilty of a sham-PVI vs. PVI trial-

the Pilot-PvP-study

Sehr geehrte Patientin, sehr geehrter Patient,

wir möchten Sie fragen, ob Sie bereit sind, an der nachfolgend beschriebenen Studie teilzunehmen.

Studien sind notwendig, um Erkenntnisse über die Wirksamkeit diagnostischer und therapeutischer Verfahren zu erhalten. Die Studie, die wir Ihnen hier vorstellen, wurde – wie es das Gesetz verlangt – von der zuständigen Ethikkommission zustimmend bewertet. Diese Studie wird sowohl in der Klinik und Poliklinik für Kardiologie des Universitätsklinikums Leipzig als auch in der Abteilung für Rhythmologie des Herzzentrums Leipzig durchgeführt.

Ihre Teilnahme an dieser Studie ist freiwillig. Sie werden in diese Studie also nur dann einbezogen, wenn Sie dazu schriftlich Ihre Einwilligung erklären. Sofern Sie nicht an der Studie teilnehmen oder später aus ihr ausscheiden möchten, erwachsen Ihnen daraus keine Nachteile.

Sie wurden bereits auf die geplante Studie angesprochen. Der nachfolgende Text soll Ihnen die Ziele und den Ablauf erläutern. Anschließend wird ein Prüfarzt das Aufklärungsgespräch mit Ihnen führen. Bitte zögern Sie nicht, alle Punkte anzusprechen, die Ihnen unklar sind. Sie werden danach ausreichend Bedenkzeit erhalten, um über Ihre Teilnahme zu entscheiden.

**1. Warum wird diese Studie durchgeführt?**

Sie leiden an Vorhofflimmern, welches mit einer Ablation behandelt werden soll. Die Ablation oder auch Pulmonalvenenisolation ist eine häufig durchgeführte und sichere Behandlung, um die Beschwerden, welche durch Vorhofflimmern verursacht werden, zu lindern. Besonders gut erprobt ist sie im Vergleich zu einer medikamentösen Therapie. Völlig unklar ist bislang jedoch, auf welche Art und Weise die Ablationsbehandlung zur Reduktion der Beschwerden führt. Ein etabliertes Verfahren, um eine solche Fragestellung zu beantworten ist die Überprüfung mithilfe einer sogenannten Schein- oder Placeboprozedur.

**Bei Ihnen wird in jedem Fall eine Pulmonalvenenisolation durchgeführt werden, wir fragen Sie nur, ob Sie theoretisch auch bereit wären, an einer Studie mit einer Schein-Pulmonalvenenisolation teilzunehmen.**

Um diese Überprüfung bei einer großen Gruppe von Patienten durchzuführen, ist es notwendig, im Vorfeld einen Überblick über möglicherweise geeignete Patienten zu erhalten. Darum möchten wir alle Patienten, die eine Ablationsbehandlung des Vorhofflimmerns erhalten, befragen, ob sie grundsätzlich bereit wären, an so einer Studie teilzunehmen. Zusätzlich möchten wir die Einschränkungen im täglichen Leben sowie die Lebensqualität erfassen, um feststellen zu können, welche Patienten vielleicht besonders geeignet für eine Teilnahme an der Studie sind.

**2. Kann es sein, dass bei mir eine Scheinprozedur durchgeführt wird?**

Nein, wir möchten nur mithilfe eines Fragebogens erheben, ob und wie sehr Sie durch das Vorhofflimmern beeinträchtigt sind und ob Sie grundsätzlich bereit wären, an einer Studie teilzunehmen, in der die Möglichkeit besteht, dass Sie eine Scheinprozedur erhalten.

**3. Wie ist der Ablauf der Studie und was muss ich bei Teilnahme beachten?**

Sie erhalten vor der geplanten Ablationsbehandlung einen Fragebogen. Wir bitten Sie, diesen wahrheitsgemäß auszufüllen. Anschließend erhalten Sie wie geplant die für Sie vorgesehene Behandlung. Wir erheben zusätzlich die Daten, welche die Behandlung betreffen (z.B. die Dauer und die verwendeten Medikamente) sowie die Ergebnisse der im Rahmen der Routine durchgeführten Untersuchungen (Laborergebnisse, Daten des Herzultraschalls, MRTs oder CTs).

Ungefähr 3 Monate nach der Ablationsbehandlung kontaktieren wir Sie telefonisch und erkundigen uns nach Ihrem Befinden. Außerdem führen wir eine kurze Befragung durch.

**4. Welchen persönlichen Nutzen habe ich von der Teilnahme an der Studie?**

Ein persönlicher Nutzen der Teilnahme ist spekulativ und kann Ihnen nicht versprochen werden.

**5. Welche Risiken sind mit der Teilnahme an der Studie verbunden?**

Wir führen nur eine Befragung durch, diese ist frei von Risiken.

**6. Wer darf an dieser Studie nicht teilnehmen?**

Alle Patienten, die für eine Pulmonalvenenisolation vorgesehen sind, dürfen an dieser Studie teilnehmen.

**7. Entstehen für mich Kosten durch die Teilnahme an der Studie? Erhalte ich eine Aufwandsentschädigung?**

Durch Ihre Teilnahme an dieser Studie entstehen für Sie keine zusätzlichen Kosten. Sie erhalten keinen finanziellen Ausgleich für Ihre Teilnahme an dieser Studie.

**8. Bin ich während der Studie versichert?**

Nein, es besteht keine Versicherung, da es sich um eine reine Befragung handelt~~.~~

**9. Werden mir neue Erkenntnisse während der Studie mitgeteilt?**

Sie werden über neue Erkenntnisse, die in Bezug auf diese Studie bekannt werden und die für Ihre Bereitschaft zur weiteren Teilnahme wesentlich sein können, informiert. Auf dieser Basis können Sie dann Ihre Entscheidung zur weiteren Teilnahme an dieser Studie überdenken.

**10. Wer entscheidet, ob ich aus der Studie ausscheide?**

Sie können jederzeit, auch ohne Angabe von Gründen, Ihre Teilnahme beenden, ohne dass Ihnen dadurch irgendwelche Nachteile bei Ihrer medizinischen Behandlung entstehen.

Unter gewissen Umständen ist es aber auch möglich, dass der Prüfarzt oder der Studienleiter entscheidet, Ihre Teilnahme an der Studie vorzeitig zu beenden, ohne dass Sie auf die Entscheidung Einfluss haben. Die Gründe hierfür können z. B. sein:

- Ihre weitere Teilnahme an der Studie ist ärztlich nicht mehr vertretbar;
- es wird die gesamte Studie abgebrochen.

Sofern Sie sich dazu entschließen, vorzeitig aus der Studie auszuscheiden, oder Ihre Teilnahme aus einem anderen der genannten Gründe vorzeitig beendet wird, bitten wir Sie, für eine abschließende Untersuchung Ihren Arzt aufzusuchen. Wenn Sie dies nicht tun, entstehen Ihnen in keiner Weise Nachteile. Bereits erhobene Daten werden innerhalb der Studie weiter ausgewertet, neue Daten werden nicht erhoben.

**11. Was geschieht mit meinen Daten?**

Während der Studie werden medizinische Befunde und persönliche Informationen von Ihnen erhoben und in der Prüfstelle in Ihrer persönlichen Akte niedergeschrieben oder elektronisch gespeichert. Die für die Studie wichtigen Daten werden zusätzlich in pseudonymisierter Form gespeichert, ausgewertet und gegebenenfalls weitergegeben.

Pseudonymisiert bedeutet, dass keine Angaben von Namen oder Initialen verwendet werden, sondern nur ein Buchstaben-/Nummerncode ohne Bezug zu Ihrer Person*.* Diese Daten werden auf einem Rechner im Universitätsklinikum Leipzig gespeichert.

Alle schriftlichen und elektronischen Studienunterlagen werden mindestens zehn Jahre nach Beendigung dieser Studie aufbewahrt. Die Ergebnisse der medizinischen Untersuchungen und der Befragung werden ausschließlich in anonymisierter Form zusammengestellt. Die Ergebnisse dieser Studie können bei Präsentationen oder in Zeitschriften vorgestellt werden, Ihre Identität wird jedoch nicht bekannt gegeben. Das bedeutet, niemand kann später aus den Ergebnissen erkennen, von welcher Person die Angaben gemacht worden sind bzw. welcher Messwert zu welcher Person gehört. Eine Löschung der anonymisierten Daten ist nicht vorgesehen.

Sie haben nach Datenschutzgrundverordnung folgende Rechte **bezüglich ihrer personenbezogenen Daten**:

**Recht auf Auskunft** über Ihre personenbezogenen Daten, die im Rahmen der klinischen Prüfung erhoben, verarbeitet oder ggf. an Dritte übermittelt werden. Sie haben hierbei Anspruch auf eine kostenfreie Kopie ihrer Daten.

**Recht auf Berichtigung** unrichtiger personenbezogener Daten.

**Recht auf Löschung** Ihrer personenbezogenen Daten mit der oben genannten Einschränkung. Recht auf Einschränkung der Verarbeitung. Die Einschränkung der Verarbeitung muss beim Prüfer oder dem Datenschutzbeauftragten des Prüfzentrums beantragt werden.

**Recht auf Datenübertragung** der zu Ihrer Person erhobenen Daten. Diese Daten werden entweder Ihnen selbst oder, soweit technisch möglich, einer anderen von Ihnen benannten Stelle übermittelt.

**Recht auf Widerruf (Datenschutzrechtliches Widerrufsrecht):** Sie haben das Recht, ohne Angabe von Gründen die Einwilligung zur Verarbeitung ihrer personenbezogenen Daten zu widerrufen (Art. 7 Abs. 3 EU-DSGVO). Die Rechtmäßigkeit der bis zum Widerruf erfolgten Verarbeitung ihrer personenbezogenen Daten wird dadurch nicht berührt.

Wollen Sie von den genannten Rechten Gebrauch machen, kontaktieren Sie bitte Ihren Prüfarzt. Bei Anliegen zur Datenverarbeitung und zur Einhaltung der datenschutzrechtlichen Anforderungen können Sie sich auch an folgende Datenschutzbeauftragte wenden:

Datenschutzbeauftragter des Studienleiters:

Datenschutzbeauftragter Hochschulbereich und Medizinische Fakultät
Augustusplatz 10 (Neues Augusteum)
04109 Leipzig

Telefon: 0341/97-30081
Fax: 0341/97-33370
E-Mail: dsb@uni-leipzig.de

Bitte wenden Sie sich im Regelfall an den Prüfer bzw. den Datenschutzbeauftragten des Prüfzentrums, denn allein das Prüfzentrum kann aufgrund des Pseudonymisierungsprozesses vollumfänglich auf Ihre Daten zugreifen bzw. entsprechende Auskünfte geben. Der Datenschutzbeauftragte des Sponsors kann vor diesem Hintergrund nur sehr begrenzt helfen.

Sie haben ein Beschwerderecht bei den Aufsichtsbehörden. Eine Liste der Aufsichtsbehörden in Deutschland finden Sie unter:

https://www.bfdi.bund.de/DE/Infothek/Anschriften_Links/anschriften_links-node.html

**Einzelheiten, insbesondere zur Möglichkeit eines Widerrufs, entnehmen Sie bitte der Einwilligungserklärung, die im Anschluss an diese Patienteninformation abgedruckt ist.**

S2: Patient information of the survey assessing patients’ attitude towards a sham-controlled trial on pulmonary vein isolation in atrial fibrillation
